# Testing a Biobehavioral Model of Chronic Stress and Weight Gain in Young Children (Family Stress Study): Protocol and Baseline Demographics for a Prospective Observational Study

**DOI:** 10.2196/48549

**Published:** 2024-06-20

**Authors:** Shannon M Pare, Elizabeth Gunn, Katherine M Morrison, Alison L Miller, Alison M Duncan, Andrea C Buchholz, David W L Ma, Paul F Tremblay, Lori Ann Vallis, Nicola J Mercer, Jess Haines

**Affiliations:** 1 Department of Family Relations and Applied Nutrition University of Guelph Guelph, ON Canada; 2 Centre for Metabolism, Obesity & Diabetes Research Department of Pediatrics McMaster University Hamilton, ON Canada; 3 Department of Health Behaviour and Health Education University of Michigan School of Public Health Ann Arbor, MI United States; 4 Department of Human Health and Nutritional Sciences University of Guelph Guelph, ON Canada; 5 Department of Psychology University of Western Ontario London, ON Canada; 6 Wellington-Dufferin-Guelph Public Health Guelph, ON Canada

**Keywords:** stress, child, preschool, adiposity, household chaos, cortisol, COVID-19, behavioral mechanisms, caregiver-child relationship quality

## Abstract

**Background:**

Chronic stress is an important risk factor in the development of obesity. While research suggests chronic stress is linked to excess weight gain in children, the biological or behavioral mechanisms are poorly understood.

**Objective:**

The objectives of the Family Stress Study are to examine behavioral and biological pathways through which chronic stress exposure (including stress from COVID-19) may be associated with adiposity in young children, and to determine if factors such as child sex, caregiver-child relationship quality, caregiver education, and caregiver self-regulation moderate the association between chronic stress and child adiposity.

**Methods:**

The Family Stress Study is a prospective cohort study of families recruited from 2 Canadian sites: the University of Guelph in Guelph, Ontario, and McMaster University in Hamilton, Ontario. Participants will be observed for 2 years and were eligible to participate if they had at least one child (aged 2-6 years) and no plans to move from the area within the next 3 years. Study questionnaires and measures were completed remotely at baseline and will be assessed using the same methods at 1- and 2-year follow-ups. At each time point, caregivers measure and report their child’s height, weight, and waist circumference, collect a hair sample for cortisol analysis, and fit their child with an activity monitor to assess the child’s physical activity and sleep. Caregivers also complete a web-based health and behaviors survey with questions about family demographics, family stress, their own weight-related behaviors, and their child’s mental health, as well as a 1-day dietary assessment for their child.

**Results:**

Enrollment for this study was completed in December 2021. The final second-year follow-up was completed in April 2024. This study’s sample includes 359 families (359 children, 359 female caregivers, and 179 male caregivers). The children’s mean (SD) age is 3.9 years (1.2 years) and 51% (n=182) are female. Approximately 74% (n=263) of children and 80% (n=431) of caregivers identify as White. Approximately 34% (n=184) of caregivers have a college diploma or less and nearly 93% (n=499) are married or cohabiting with a partner. Nearly half (n=172, 47%) of the families have an annual household income ≥CAD $100,000 (an average exchange rate of 1 CAD=0.737626 USD applies). Data cleaning and analysis are ongoing as of manuscript publication.

**Conclusions:**

Despite public health restrictions from COVID-19, the Family Stress Study was successful in recruiting and using remote data collection to successfully engage families in this study. The results from this study will help identify the direction and relative contributions of the biological and behavioral pathways linking chronic stress and adiposity. These findings will aid in the development of effective interventions designed to modify these pathways and reduce obesity risk in children.

**Trial Registration:**

ClinicalTrials.gov NCT05534711; https://clinicaltrials.gov/study/NCT05534711

**International Registered Report Identifier (IRRID):**

DERR1-10.2196/48549

## Introduction

Chronic stress is increasingly recognized as an important risk factor in the development of obesity [1]. Among children aged as young as 2-6 years, exposure to chronic stress, including parental divorce, parental depression, and household chaos has been associated with higher weight status and excess weight gain over time [2-4]. The COVID-19 pandemic fundamentally disrupted life for families and has been a key source of chronic stress for young children [5,6] and their parents [7].

While research suggests chronic stress is linked to excess weight gain in children, the biological or behavioral mechanisms are poorly understood [8]. Biologically, a central aspect of the stress response is the activation of the hypothalamic-pituitary-adrenal axis, leading to increased circulating cortisol levels [9]. Prolonged elevation of cortisol, a glucocorticoid, leads to increased adiposity, particularly abdominal adiposity, as well as increased appetite and preferences for high-fat and high-sugar foods [9]. Few studies have examined the association between cortisol and obesity among young children and most of this research has been cross-sectional [10-12]. Biological stress could be implicated in weight gain through complex pathways that develop over time; thus, longitudinal research is needed to elucidate the direction of these pathways [8]. Putative behavioral mechanisms linking stress with excess weight gain include consumption of energy-dense foods [13], eating for comfort [14], low physical activity [15], high screen time [16], and poor sleep [17]. However, research examining these mechanisms over time is sparse, particularly among young children [8]. Understanding these behavioral mechanisms, including the relative contributions of weight-related behaviors linking family stress and excess weight gain in children, can help inform behavioral targets for obesity interventions for young children who experience stress in the home.

In previous studies, researchers concluded that the damaging impact of stress can be buffered when children have supportive relationships with their caregivers [18-20]. The ability of parents to manage their own emotions during times of stress, that is, parental self-regulation, also influences their child’s stress response [21]. Epidemiologic [3] and laboratory [22] research suggests that, when exposed to similar stressors, females have stronger biological reactivity to stress compared to males. Further, low-income households and households with less educated parents are associated with higher-stress environments [23,24] and children from these families bear a disproportionate share of the burden of obesity [25]. These findings underscore the need to examine how child sex, caregiver-child relationship quality, and caregiver self-regulation and education may moderate the pathways linking stress and excess weight gain in children.

**Figure 1:**
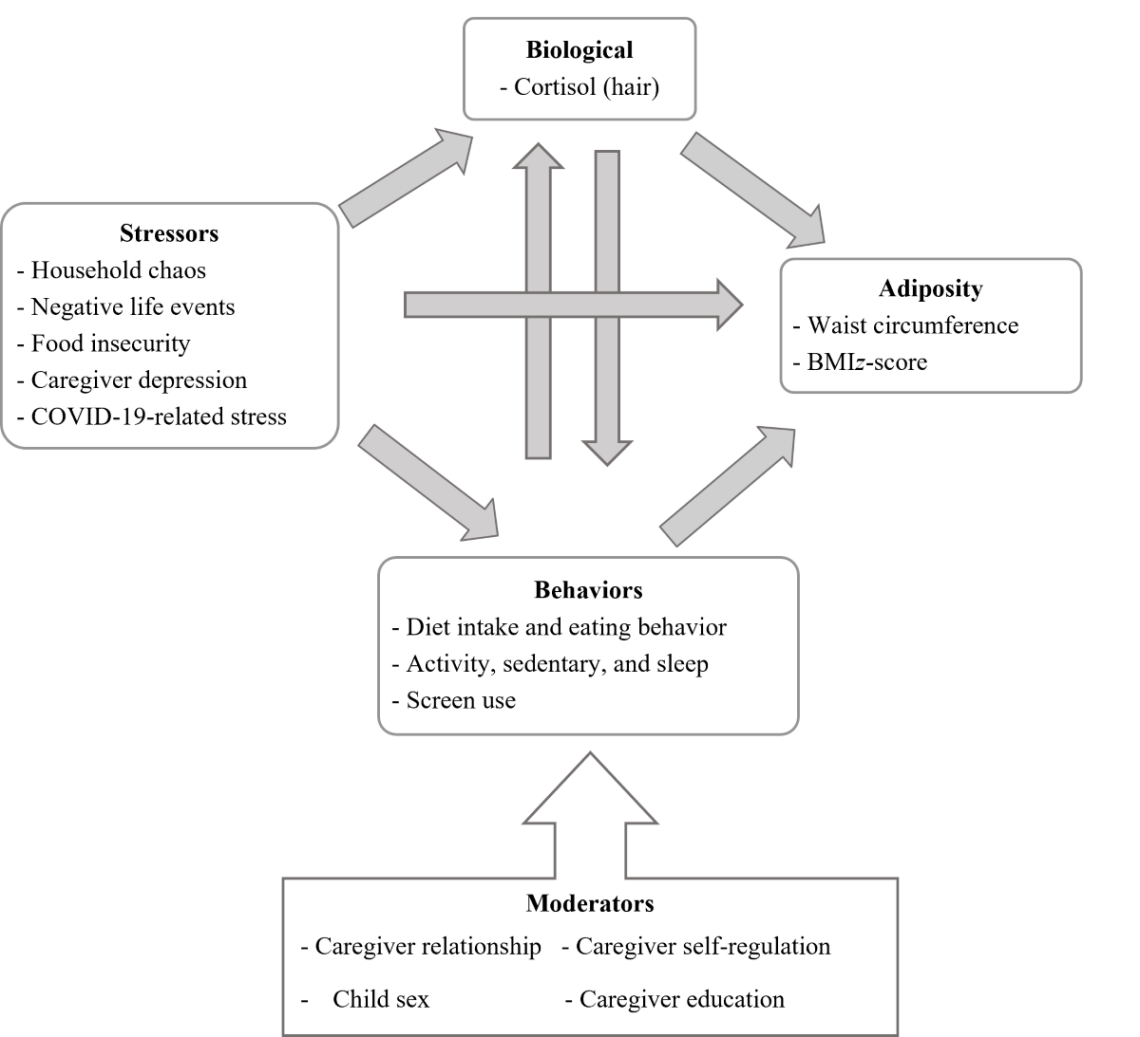
Conceptual model for the Family Stress Study: a prospective observational study of biological and behavioral pathways linking stress and child adiposity in young children.

Building on this existing research, the primary objective of the Family Stress Study is to test the conceptual model (Figure 1), using longitudinal structural equation modeling (SEM), that exposure to chronic stressors is directly associated with higher adiposity among children and partly mediated through alterations in children’s cortisol production and weight-related behaviors. It is hypothesized that (1) chronic stress at baseline will be positively associated with children’s BMI *z* score and waist circumference (WC) at 1- and 2-year follow-up, (2) the association between chronic stress and children’s adiposity outcomes will be partially mediated by children’s cortisol levels and children’s weight-related behaviors, and (3) associations between children’s cortisol levels and weight-related behaviors will be bidirectional (modeled as a cross-lagged structure within the longitudinal design). The secondary objective of this study is to examine the extent to which child sex, caregiver-child relationship quality, caregiver education, and caregiver self-regulation, moderate the associations examined in objective 1. Based on previous research, it is hypothesized that associations between chronic stress, cortisol levels, weight-related behaviors, and adiposity will be stronger among (1) children with less supportive caregiver relationships as compared to those with more supportive relationships [18,19], (2) female children as compared to male children [3,22], (3) children whose caregivers have lower education as compared to children whose caregivers have higher education [23,24], and (4) children whose caregivers have lower self-regulation as compared to children whose parents have higher self-regulation [21].

The variables depicted in Figure 1 will be assessed across 3 time points, measured 1 year apart, which will allow the elucidation of the direction and the relative contributions of the complex biological and behavioral pathways linking chronic stress and adiposity. Study results will help inform interventions to modify these pathways and reduce obesity risk among young children in the post–COVID-19 context. This paper describes the approach, protocol methods, and baseline demographics of the participants enrolled in the Family Stress Study.

## Methods

### Study Design

**Figure 2:**
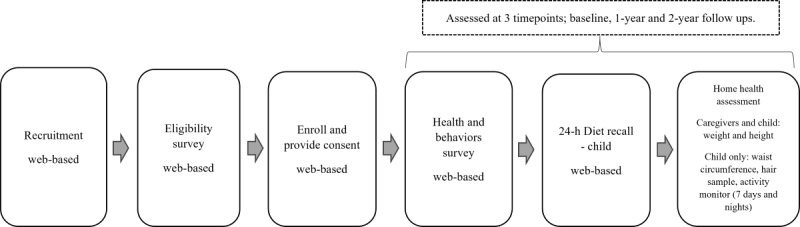
Design of the Family Stress Study for remote recruitment and data collection to test a biobehavioral model of chronic stress and weight gain in young children.

The Family Stress Study is a prospective cohort study of children aged 2-6 years recruited from 2 Canadian sites: the University of Guelph in Guelph, Ontario, and McMaster University in Hamilton, Ontario. Ethics approval was obtained before the restrictions due to the COVID-19 pandemic were enacted in Ontario in March 2020, but recruitment had not begun. The original protocol, which included an in-person health assessment of adiposity using air displacement (bod pod) and researcher-led measures of child and caregiver height, weight, and child WC, was revised to a caregiver-led at-home health assessment of child and caregiver weight, height, and child WC to adhere to the COVID-19 safety protocols. Measures of COVID-19–related stress were added to the web-based health and behaviors survey. The revised protocol was approved by both research ethics boards. Figure 2 provides an overview of this study’s design.

### Study Participants and Recruitment

Between July 2020 and December 2021, participants were recruited from the city of Guelph, the city of Hamilton, and surrounding areas. Recruitment was conducted primarily via the web using paid advertisements on Facebook (Meta) and Instagram (Instagram from Meta), and social media posts, including posts by community organizations that serve families, and through posters in community locations, including libraries and toy stores. All methods of recruitment provided caregivers with study contact information and directed them to this study’s website, where caregivers obtained additional details regarding this study’s process and interested caregivers completed an eligibility screener.

To be eligible to participate, families had to have at least one child aged 2-6 years and have no plans to move from the area within the next 3 years. Participants were excluded if they were (1) enrolled in the Guelph Family Health Study (a longitudinal study led by the investigative team) at the University of Guelph to allow data to be merged across the 2 studies; (2) children born preterm (before 34 weeks gestation); (3) children with any health condition expected to affect cortisol production, obesity, or growth or body composition (eg, Prader Willi syndrome and Cushing disease); and (4) children taking steroid medications, including oral or inhaled corticosteroids, medications for attention-deficit/hyperactivity disorder, or antidepressants or antipsychotic medications.

To participate, caregivers had to live with the child at least 50% of the time and could include parents (biological, related, adoptive, or foster), grandparents, aunts, and uncles. The primary caregiver was the caregiver who first signed up for this study and served as the main contact for the family. Eligible primary caregivers completed a web-based consent to enroll themselves and their oldest eligible child in the target age range (2-6 years). Primary caregivers provided contact information for themselves and, if applicable, an email address for a second caregiver. The secondary caregiver was contacted separately to enroll and complete web-based consent. Study coordinators also connected regularly with families via email, text, or phone to provide them with support throughout the data collection process.

### Ethical Considerations

This study was approved on March 11, 2020, and the amended protocol for remote data collection was approved by the University of Guelph Research Ethics Board (11-19-047) on June 12, 2020. The Hamilton Integrated Research Ethics Board approved this study on July 7, 2020, along with approval for the amended protocol on July 20, 2020 (10763). Recruitment and data collection began in July 2020 (Guelph) and August 2020 (Hamilton); primary caregivers enrolled in this study provided informed consent for themselves and their oldest eligible child and secondary caregivers provided informed consent for themselves. All participant data will be deidentified and only group data will be presented in all reports of this study’s results. At each data collection time point, participating families receive a CAD $25 grocery gift card per caregiver for completing a health and behaviors survey (also includes the diet recall for the child), and a CAD $30 grocery gift card per family for completing the at-home health assessment as a thank you for their time.

### Data Collection

Chronic stress and children’s weight-related behaviors including diet and physical activity or sleep, cortisol levels, and adiposity are assessed at baseline, 1- and 2-year follow-ups. Further, 1-year intervals for assessment are based on Canadian health practice guidelines that recommend measuring height and weight once per year for children aged 2 years and older [26]. At each time point, primary caregivers complete a web-based health and behaviors survey with questions about family demographics, family stress, child weight-related behaviors, mental health, and their own weight-related behaviors. Secondary caregivers complete an abbreviated version of the health and behaviors survey focused on measures of stress and their own weight-related behaviors. Primary caregivers complete a web-based 1-day dietary assessment for their child. Caregivers complete anthropometric measures, collect a hair sample for cortisol analysis, and fit their child with an activity monitor for the at-home health assessment.

### Measures

#### Child Adiposity

Families are provided with the necessary materials (a nonelastic flexible tape measure and Seca 803 digital scale [Seca]) and detailed, illustrated instructions [27-29] to complete the at-home health assessment, including the child’s WC [30], height [31], and weight. Caregivers report the measurements using a web-based survey. This process has been validated among preschool-aged children in previous studies [27-29]. BMI *z* scores are calculated in R^22^ using the “zscorer” package, based on the World Health Organization child growth standards [32]. WCs are measured by a caregiver to the nearest 0.1 cm using a soft retractable tape measure at the top of the iliac crest as per the protocol in the Canadian Health Measures Survey [30].

#### Child Hair Cortisol

Hair samples are used to assess children’s chronic cortisol concentrations as an indicator of chronic biological stress. Primary caregivers are instructed to cut a lock of hair approximately 1 cm in width from the posterior vertex [33], as close as possible to the scalp, and secure it to a collection card with the scalp end identified. Using hair to assess cortisol is more reproducible than other physiological measures, for example, saliva, because it aggregates day-to-day variations in hypothalamic-pituitary-adrenal axis activity, thereby reflecting chronic rather than acute stressful exposures [34,35]. Hair samples are sent for cortisol analysis at the Centre for Studies on Human Stress, Montreal, Quebec, a laboratory focused on analyzing stress hormones.

The Centre for Studies on Human Stress analyzes 3 cm of hair, representing approximately 3 months of stress exposure [36]. In brief, a 25 mg sample of hair is placed in a vial to which 2.5 mL of isopropanol is added and mixed for 3 minutes on a plate rotator. This wash procedure is repeated once, and hair samples dry overnight. Then, the washed hair is transferred to a new vial to which 1.5 mL of pure methanol is added and vials are rotated for 24 hours. Samples are spun in a centrifuge for 2 minutes at 10,000 rpm and 1 ml of the clear supernatant is transferred into a new vial. Methanol is then evaporated under a constant stream of nitrogen at 60°C for 20 minutes or until dry. When dried, 0.4 mL of phosphate buffer (CAL A, IBL-Hamburg) is added to the tube, and then vortexed for 15 seconds [37]. A commercially available immunoassay with chemiluminescence detection from IBL-International is used to determine cortisol concentrations. The ranges of assays at 0.015-3.20 µg/dL are converted to picograms or milligrams for reporting.

### Chronic Stress

Chronic stress is assessed using 5 different measures of stress: household stress, food insecurity, caregiver depression, children’s negative life events, and COVID-19–related stress. Household stress is quantified using the 15-item Confusion, Hubbub, and Order Scale (CHAOS) [38]. Primary caregivers respond to items including “you can’t hear yourself think in our home” or “there is very little commotion in our home” on a 4-point Likert scale from 1 (very much like your own home) to 4 (not at all like your own home). After reverse scoring relevant items, a total score will be calculated by summing the responses. The full CHAOS score [39-41], as well as the Emotional CHAOS subscale [4], created using 8 of the full-scale items, have been associated with obesity risk and obesogenic eating behaviors among young children. Standardized Cronbach α for the CHAOS survey was found to be 0.78 in the Guelph Family Health Study [42], which is a longitudinal study of families with toddlers and preschoolers [43].

Food security is examined using the Household Food Security Survey Module, an 18-item measure that includes 10 items that assess uncertain, insufficient, or inadequate food access related to adults in the household and 8 items focused on children over the previous 12 months [44]. Households will be categorized based on the total number of positive responses: food secure households (0 responses), marginally food insecure households (1 responses), moderately food insecure households (2 to 5 responses), or severely food insecure households (≥6 responses). While evidence for an association between food insecurity and obesity risk in children has been mixed [45], food insecurity is a prevalent source of stress among families, affecting 1 in 6 Canadian children [46], and has been shown to be associated with poorer diets in children [47].

Caregiver depressive symptoms are assessed using the 20-item short-form Centre for Epidemiologic Studies Depression Scale (CES-D) [48-50]. Both the primary and second caregivers respond to statements about the previous week including “everything I did was an effort” and “I felt lonely.” Responses are to be scored as 0 (less than 1 day), 1 (1-2 days), 2 (3-4 days), or 3 (5-7 days) and, after reverse scoring relevant items, a total score out of 30 will be calculated by summing the responses. The standardized Cronbach α for the CES-D was found to be 0.88 for mothers and 0.80 for fathers of young children in the Guelph Family Health Study [51]. Parental depression assessed using CES-D has been found to be associated with obesity risk and obesity-related behaviors in children [51,52].

Children’s negative life events are reported using an abbreviated List of Threatening Experiences [53], an 8-item measure of negative life events, for example, divorce. Primary caregivers report negative events for their child across their lifetime (or for the past year on the follow-up surveys) by answering “yes” or “no” to whether or not their child has encountered various events, such as “divorce or separation of parents” or “severe disease or accidents.” The total score will be determined by summing the number of negative events. This measure has been found to be associated with obesity risk in children [54].

COVID-19–related stress is assessed by asking both the primary and secondary caregiver about quarantine or illness due to COVID-19, change in employment status due to COVID-19 (eg, loss of job and change in work hours per location), and employment in essential services (eg, first responder, health care worker, critical infrastructure worker, food production and supply, production, supply of medicine, hydro, and gas, and corrections). Given that COVID-19 disrupted home and work life among families with young children, measures assessing work conflict [55], financial insecurity [56], and parenting stress [57] are also included in the caregiver’s web-based survey. Work-to-family conflict is assessed using a 5-item scale adapted from Carlson et al [55]. Caregivers are asked questions including “my work keeps me from family activities more than I would like” and “when I am home from work, I am often too frazzled to participate in family activities or responsibilities.” Responses are on a 5-point Likert scale from 1 (strongly disagree) to 5 (strongly agree) [55]. A total score will be calculated by summing the responses.

Financial insecurity is evaluated using 2 items adapted from the measures by Gundersen and Boushey [56]: “during the past month, was there a time when you were worried you would not be able to pay the mortgage, rent or other bills on time?” and “are you worried about not being able to pay the mortgage, rent or other bills on time over the next 6 months?” to which caregivers respond “yes” or “no.” Families will be coded as financially insecure if they respond positively to either item.

Parenting stress is assessed by asking caregivers 12 items from the parenting distress domain of the short-form parenting index [58]. Items include “I feel trapped by my responsibilities as a parent/caregiver,” “there are quite a few things that bother me about my life,” and “having a child has caused more problems than I expected in my relationship with my spouse/partner (or male/female friend).” Responses are on a 5-point Likert scale from 1 (strongly disagree) to 5 (strongly agree). After reverse scoring relevant items, a total score will be calculated by summing the responses. The standardized Cronbach α in a similar sample was 0.86 among mothers and 0.78 among fathers [59].

### Child Weight-Related Behaviors

#### Dietary Intake and Diet Quality

Primary caregivers report their child’s dietary intake for 1 day using the web-based Automated Self-Administered 24-hour Dietary Assessment Tool (ASA24)-Canada. ASA24 includes multiple prompts for participants to facilitate accurate data entry. A validity study that compared parent reports to children’s true diet intake among 40 parent-child dyads found that parents could accurately report what young children ate and drank [60]. ASA24-Canada analyzes the dietary data using the Canadian Nutrient File and a Health Canada recipe database along with the United States Food and Nutrient Database for Dietary Studies and the Food Patterns Equivalents Database. These databases enable ASA24-Canada to output a summary of the food descriptions, energy and nutrient intakes, and United States Department of Agriculture Food Pattern components. This information will be used to examine children’s intake based on evidence that stress is associated with a higher intake of fast food [61,62], high-fat and high-sugar foods [13], and lower intake of fruit and vegetables [13].

#### Eating Behavior

Further, 4 eating behaviors that are most consistently associated with increased obesity risk in children (enjoyment of food [4 items], food responsiveness [5 items], emotional overeating [4 items], and satiety responsiveness [5 items]) [3,63,64] are assessed using the Children’s Eating Behavior Questionnaire [65]. Parents report their children’s eating behavior for each of these behaviors, for example, “my child eats more when anxious.” Response options for each item include “never” (1), “rarely” (2), “sometimes” (3), “often” (4), and “always” (5). After reverse coding relevant items, a mean score will be calculated for eating behavior with higher scores reflecting higher endorsement of these behaviors. The Children’s Eating Behavior Questionnaire subscales have been shown to have good internal consistency (Cronbach α ranging from 0.72 to 0.91) when tested among a sample of families with young children [65].

#### Physical Activity, Sedentary Behavior, Sleep, and Screen Time

A lightweight activity monitor (wGT3x-BT; Actigraph; 100 Hz) with idle sleep mode enabled to preserve battery life, is fixed to a soft strap and placed on the child’s right hip for 7 consecutive days, 24 hours/day, and the monitors are returned via prepaid mail or picked up from participant’s homes. To aid with compliance, caregivers are provided with a link to an animated video to share with their child about the activity monitor and why they are being asked to wear it. Caregivers complete a log sheet to track monitor wear-time, including times that the device is removed (ie, bathing) in addition to their child’s sleep and wake times. Using validated age-appropriate cut-point values [66], data collected from the monitors will then be used to quantify physical activity, that is, total physical activity and moderate to vigorous physical activity, total sedentary time [67], and total sleep [68,69].

Primary caregivers report child screen time defined as “any time that is spent on screens such as televisions, cell phones, iPads or tablets and video games” on a typical weekday and weekend day over the past week [70]. Weekday and weekend day values will be summed and averaged to calculate the average daily screen time.

### Potential Moderating Variables

Primary caregivers report their child’s sex. Both the primary and secondary caregiver report their education level. Quality of caregiver relationship is assessed for both primary and secondary caregivers using the warmth or affection subscale of the Parenting Acceptance and Rejection Questionnaire [58,71] which measures parents’ feelings of warmth and acceptance (vs rejection) toward their child, a central aspect of relationship quality [72,73] that has been associated with multiple child outcomes [74,75]. Caregivers respond to items including “I say nice things about my children” on a 4-point Likert scale from 1 (almost always true) to 4 (almost never true). After reverse coding relevant items, a total score will be calculated by summing the responses. Both the primary and secondary caregivers completed an abbreviated 30-item version of the Behavior Rating Inventory of Executive Function [76] to assess caregiver self-regulation. A total score will be calculated by summing the responses across the 30 items. Time-varying moderators will be assessed at all 3 time points, whereas time-invariant moderators will only be assessed at baseline.

### Covariates

Primary caregivers report their child’s birth weight, gestational age, duration of breastfeeding, annual and sources of household income, child and caregiver race or ethnicity, and caregiver employment status (full- or part-time). Secondary caregivers report their race or ethnicity and their employment status.

### Retention

The research team has extensive experience in strategies for retention in longitudinal family studies [77-79]. To maximize retention in the Family Stress Study, many of these same strategies are used including (1) regular emails to maintain connection with participants, (2) mailings with tracking to obtain forwarding address information, and (3) financial incentives for assessments.

### Data Analyses

To summarize baseline caregiver and child characteristics for this paper, descriptive statistics were used. Percentages are presented for categorical data. Means and SDs are used for continuous data.

This study’s objectives will be tested using an SEM approach for longitudinal models that combines latent growth models (LGMs) with auto-regressive and cross-lagged paths using a special parameterization that separates the time-invariant (between-individuals) and time-varying (within-individuals) components [80,81]. Before constructing the SEM models, distributions of all the variables, their skewness and kurtosis levels, and their presence of extreme or outlier responses above or below 3 times the IQR will be explored and outliers will either be removed or Winsorized.

An incremental model-building approach similar to the recommended approach by Curran et al [80] will be used to test this study’s objectives. The first component in the model-building approach is the LGM of the trajectories across time for the variables’ chronic stressors (explanatory variable), child cortisol and child behavior (mediator variables), and child adiposity (outcome variable). Each variable has its own trajectory and includes an intercept (I), which refers to a baseline value, and a slope (S), which refers to change over time. Of particular importance is that individual child variation in these I and S parameters will be modeled. If any of these components are found to be not statistically significant, they are removed from the model. Typically, the I means and variances are significantly different from 0, but the S means may or may not depict significant linear change. The second component consists of the auto-regressive associations (ie, T2 regressed on T1, and T3 on T2) capturing the rank-order stability of the variables across the 3 time points (eg, the relationship between cortisol at T1 and T2). The third component typically consists of cross-lagged paths or other hypothesized paths between the variables, accounting for growth factors, and stability factors. In this study, this component will consist of the longitudinal mediation paths (ie, from explanatory variable at T1 to mediator at T2, to outcome at T3). The hypothesized model includes 2 partial mediators of the relation between stressors and child adiposity: child cortisol and behaviors. To test mediation, a direct path from stressors at T1 and child adiposity at T3 is also included. A correlational path between the residualized mediators is also included to model any leftover covariance between them. The fourth component will assess the moderation of the mediated paths. For example, to test the hypothesis that the cortisol mediation may differ across the sex of the children, we can use a multiple groups SEM approach that compares the effect size of mediation across male and female children.

As with any longitudinal study, attrition is expected. Patterns of missing data and potential bias at time 2 and time 3 will be investigated by comparing demographics and variable scores of participants who are missing to those who are not missing at each time point. In addition to noting and acknowledging any bias due to missingness, the SEM modelling procedure uses a maximum likelihood estimator which also provides one of the best ways to estimate the model parameter estimates in the presence of missing data. While there is no guarantee that the data are missing completely at random, in longitudinal designs, data from earlier time points compensates for the missingness at later time points in the estimation procedure.

### Sample Size Estimation

To estimate the power of specific paths in the model, an empirical simulation approach was used [82,83]. To run power analysis for LGM models, Muthén and Muthén [83] proposed a general framework using Monte Carlo simulation. Further, 2 separate a priori power analyses were conducted to determine whether a sample size of 300 is sufficient to detect the cross-lagged effects of interest. The first model assumed data were normally distributed and the second model assumed nonnormality in 10% (n=30) of the cases. For each simulation, a sample size of 300 was specified with 10,000 replications successfully completed. For both the normally distributed and nonnormally distributed data scenarios, all estimated parameters had between 82% and 86% coverage, and the parameter estimate and SE bias were below 5%, suggesting sufficient power.

## Results

**Figure 3:**
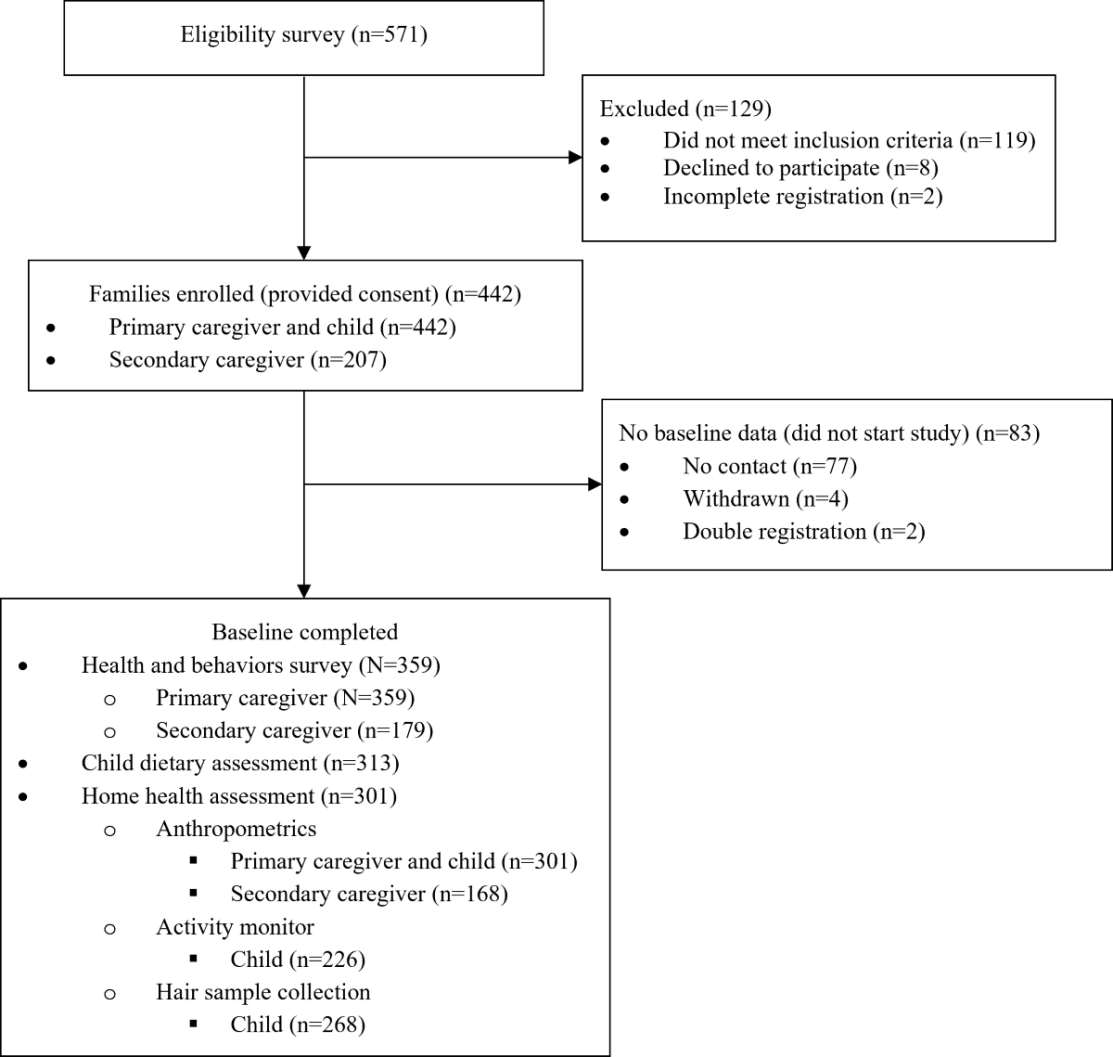
Diagram of participant flow from recruitment to collection of baseline data of the Family Stress Study (July 2020 and May 2022).

As illustrated in Figure 3, a total of 571 primary caregivers completed eligibility surveys (Guelph n=298; Hamilton n=273). Of those, 129 did not enroll in this study: 119 did not meet inclusion criteria, 8 declined to participate, and 2 were excluded for incomplete registrations. Of the 442 eligible families enrolled (ie, provided consent), 79 did not complete any baseline measures (ie, did not start this study) and 4 withdrew. Baseline health and behavior surveys were completed for 359 families including 359 primary caregivers (96% women [n=346]), 179 secondary caregivers (92% men [n=166]), and 359 children. Diet recalls were completed for 313 children and 226 children had valid activity monitor wear time (minimum of 360 min for a minimum of 3 days). A total of 301 families completed an at-home health assessment which included anthropometric measures for 301 primary caregivers, 168 secondary caregivers, and 301 children along with the collection of 268 hair samples from the children. Of the 359 families who completed baseline health and behavior surveys, 79% (n=284) identified that they heard about this study through social media, including posts by community organizations that served families and through paid Facebook advertisements.

Participant baseline characteristics are shown in Table 1 for the children and Table 2 for the caregivers. There were 182 female (50.7%) and 176 male (49%) children, with a mean (SD) age of 3.9 (1.2) years. There were 359 female and 179 male caregivers, with a mean (SD) age of 36.3 (4.8) years. Approximately 74% (n=263) of children and 80% (n=431) of caregivers identified as White. Approximately 34% (n=184) of caregivers had a college diploma or less and nearly 93% (n=499) were married or cohabiting with a partner. Nearly half (n=172, 47%) of the families had an annual household income of CAD $100,000 or more.

Enrollment for this study completed in December 2021. The final second-year follow-up was completed in April 2024. Data cleaning and analysis are ongoing.

**Table 1:** Baseline characteristics of data collected from July 2020 to May 2022 from Guelph and Hamilton, Ontario, for children (n=359) enrolled in the Family Stress Study.

Characteristics		Values (n=359)
Age (years), mean (SD)		3.9 (1.2)
**Gender, n (%)**		
	Male	179 (33.2)
	Female	359 (66.6)
**Race or ethnicity, n (%)**		
	Aboriginal or First Nations	9 (2.5)
	Black	10 (2.8)
	Chinese	8 (2.2)
	Latin American	7 (1.9)
	South Asian	11 (3.1)
	Southeast Asian	8 (2.2)
	West Asian	4 (1.1)
	White	263 (73.5)
	Not stated	5 (1.4)
	Other races or ethnicities (including mixed)	34 (9.5)

**Table 2:** Baseline characteristics of data collected from July 2020 to May 2022 from Guelph and Hamilton, Ontario, for caregivers (n=538) enrolled in the Family Stress Study.

Characteristics		Values (n=538)
Age (years), mean (SD)		3.9 (1.2)
**Sex, n (%)**		
	Male	176 (49)
	Female	182 (50.7)
**Race or ethnicity, n (%)**		
	Aboriginal or First Nations	9 (1.7)
	Black	10 (1.9)
	Chinese	12 (2.2)
	Latin American	13 (2.4)
	South Asian	20 (3.7)
	Southeast Asian	9 (1.7)
	West Asian	7 (1.3)
	White	431 (80.3)
	Not stated	6 (1.1)
	Other races or ethnicities (including mixed)	17 (3.2)
**Household income, n (%), CAD $^a^**		
	≤49,999	34 (9.5)
	50,000 to 99,999	128 (35.8)
	100,000 to 149,999	101 (27.9)
	>150,000	71 (19.8)
	Not stated	25 (7)
	Household income, median $ (IQR)	102,800 (50,000)
**Married or cohabiting, n (%)**		
	Married or cohabiting	499 (92.8)
	Not married	37 (6.9)
	Not stated	2 (0.3)
**Education, n (%)**		
	College diploma or less	184 (34.3)
	Some university or degree	161 (30.3)
	Postgraduate training	189 (35.2)
	Not stated	3 (0.6)

^a^An average exchange rate of 1 CAD=0.737626 USD applies.

## Discussion

### Principal Results

Despite public health restrictions due to COVID-19, which prevented in-person recruitment, the Family Stress Study was successful in recruiting 359 families into the Family Stress Study cohort, and 301 of those families completed all baseline measures for their child. Web-based recruitment, in particular partnering with various community organizations that serve families with young children, was critical to the recruitment success during this time when in-person engagement was limited. Identifying creative ways to connect and share information with families was also needed to ensure effective data collection. This included having research staff connect regularly with families via email, text, or phone to provide them with support throughout the data collection process and by providing information about using accelerometers to measure physical activity in a family-friendly video that could be shared remotely. These approaches were well received by families and could help inform future family-based research using remote recruitment and data collection.

### Limitations

Although successful in engaging the required sample size, the use of remote recruitment strategies may have impacted the socioeconomic diversity of the sample. While a previous family-based intervention study found that web-based recruitment of families with young children resulted in equivalent participant demographics to traditional recruitment strategies (eg, in-person or via practitioners) [84], other intervention [85] and noninterventional [86] family-based obesity studies have found that in-person recruitment was critical to engaging families, particularly low-income families. Thus, it is possible that had in-person recruitment been possible, more low-income families could have been recruited. Nearly half of the families in the sample reported an annual household income greater than CAD $100,000, which may limit the generalizability of the findings to more socioeconomically disadvantaged families. However, the median income of the sample was CAD $102,800, which is similar to the 2020 median household income (before taxes) for families in Guelph and Hamilton, which was CAD $114,000 and CAD $107,000, respectively [87]. Similarly, in our sample 73.1% (n=263) of the children identified as White, which is similar to the 2021 census data for Guelph and Hamilton which was 73.1% and 72.6%, respectively [87], suggesting that the annual household incomes and race or ethnicity of the children in our sample may be representative of the overall population within these cities.

The need to use remote data collection for the primary outcome, child adiposity, is also a limitation of the Family Stress Study. While the protocols are based on validated methods, the use of caregiver assessments may result in greater error in child adiposity measures than if using researcher-led assessments and this could lead to misclassification for some children. However, the use of caregiver-led assessments allowed the researchers to feasibly implement this study during the COVID-19 pandemic, which represented an unprecedented disruption to family life and associated increase in family stress.

### Conclusions

To design effective obesity prevention interventions, there needs to be a clear understanding of key factors influencing excess weight gain in children. By identifying both the direction and the relative contributions of the biological and behavioral pathways linking chronic stress and adiposity, results from the Family Stress Study will help inform interventions to modify these pathways and reduce obesity risk among young children. Specifically, this research will identify the key behavioral drivers linking stress and obesity to inform which weight-related behaviors should be targeted with interventions in families experiencing high stress. This research will also identify whether the quality of caregiver relationships, child sex, and caregiver education and self-regulation moderate the pathways linking stress and obesity; this information is needed to inform what types of families may be best targeted in obesity prevention interventions. Thus, the results of this research will provide a much-needed basis to inform future childhood obesity prevention interventions.

## Abbreviations

ASA24-Canada: Automated Self-Administered 24-hour Dietary Assessment Tool
CES-D: Centre for Epidemiologic Studies Depression Scale
CHAOS: Confusion, Hubbub and Order Scale
I: intercept
LGM: latent growth model
S: slope
SEM: structural equation modeling
WC: waist circumference

## Data Availability

Due to privacy concerns, the data generated and analyzed during the Family Stress Study are not publicly available as related to the research ethics board requirements.
